# Beyond anatomy: deciphering metastasis to enhance diagnosis and treatment in head and neck carcinoma of unknown primary

**DOI:** 10.3389/fonc.2026.1878089

**Published:** 2026-08-03

**Authors:** Xinyao Han, Hui Huangfu

**Affiliations:** 1 First Clinical Medical College of Shanxi Medical University, Taiyuan, Shanxi, China; 2 Department of Otolaryngology Head and Neck Surgery, The First Hospital of Shanxi Medical University, Taiyuan, Shanxi, China

**Keywords:** cervical lymph node metastasis, EMT, FDG-PET/CT, head and neck carcinoma of unknown primary, HPV, narrow band imaging, transoral robotic surgery

## Abstract

Head and Neck Carcinoma of Unknown Primary (HNCUP) denotes a condition where histologically confirmed cervical metastatic cancer lacks a discernible primary tumor site despite exhaustive evaluation. Patients typically present with incidentally detected neck masses, often without overt symptoms or with mild discomfort, with squamous cell carcinoma as the predominant histotype. The intricate interplay of tumor biology, lymphatic drainage, and molecular pathology in HNCUP poses significant challenges to the identification of the primary lesion using conventional imaging and endoscopic modalities, thereby impeding clinical diagnosis and management. This review will examine the potential mechanisms of metastasis in HNCUP, the characteristics of cervical lymph node metastasis, and recent advancements in diagnostic and therapeutic strategies. By thoroughly analyzing lymph node metastasis patterns and potential mechanisms, and by integrating modern imaging techniques with molecular marker detection, we aim to enhance the recognition rate of primary lesions and lay the groundwork for personalized treatment.

## 1 Introduction

In clinical practice, it is not uncommon for patients to present with neck masses without any identifiable primary tumors. It is essential to differentiate whether these masses are tumors originating from the head and neck or metastatic lesions from other regions. When pathology confirms metastatic cancer but the primary source remains unidentified despite thorough examination, this condition is termed HNCUP. Most cervical metastatic cancers can be traced to primary sites through standardized examinations, although a small proportion pose diagnostic challenges. Approximately 5% to 10% of head and neck malignant tumors initially manifest as HNCUP, with over 75% being squamous cell carcinoma (SCC) (1, 2). “True” HNCUP specifically denotes instances where the primary tumor site remains unidentified despite thorough imaging, endoscopic examinations, and surgical assessments (including biopsy and/or suspected primary site resection). These cases constitute merely 1% to 2% of head and neck cancers, and advancements in detection techniques may further reduce their prevalence (2). This review primarily focuses on patients who initially present with HNCUP. The histological nature of the metastatic lesion in such scenarios holds significant importance in pinpointing the primary lesion. From an anatomical perspective, the extensive lymphatic network of the head and neck predisposes tumor cells to early dissemination to regional lymph nodes, particularly the Level II upper deep cervical lymph nodes, representing a typical metastatic pattern (3).

However, certain metastatic foci display characteristics of “skipping metastasis” or “atypical distribution,” indicating that their metastasis patterns are not solely reliant on anatomical pathways. Instead, these patterns may involve dynamic biological processes such as phenotypic plasticity, epithelial-mesenchymal transition (EMT), and clonal selection of circulating tumor cells (CTCs)—concepts well-established in head and neck squamous cell carcinoma (HNSCC) and general cancer biology that provide a plausible framework for understanding HNCUP dissemination, though direct HNCUP-specific validation remains limited (4, 5). It is important to emphasize that EMT, CTC biology, and “seed and soil” hypothesis represent established concepts in broader oncology and HNSCC research. We apply these established frameworks to the HNCUP context to construct a conceptual model that may explain the characteristic early metastatic dissemination and occult primary phenotype observed in this disease. Nevertheless, we do not claim that these processes define a biologically distinct entity unique to HNCUP. This review examines the potential metastasis mechanisms of HNCUP, the metastatic patterns of cervical lymph nodes, and the latest advancements in diagnostic and treatment strategies. Understanding these mechanisms in HNCUP is crucial for identifying primary tumors and represents an important area for future HNCUP-specific translational research.

## 2 Potential mechanisms and metastasis patterns of HNCUP

### 2.1 Epithelial-mesenchymal transition

HNCUP is a type of malignant tumor that poses significant challenges in clinical diagnosis. Its core pathological feature is not the absence of the primary lesion, but rather the robust early invasive and disseminating potential consistent with EMT-driven progression, which leads to the regional colonization of the head and neck by cancer cells before they can be detected by imaging. In some cases, the primary lesion may even completely regress due to immune clearance or spontaneous apoptosis, presenting a “cryptic” phenotype (6, 7). The biological essence of this phenomenon lies in the high plasticity, migratory capacity, invasive potential, and microenvironmental adaptability that EMT confers on cancer cells.

In HNSCC models, the initiation of EMT follows a microenvironment-driven pattern. Cancer cells originating from mucosal sites—typically the oropharynx, nasopharynx, or hypopharynx—are subjected to chronic inflammation, hypoxia, and mechanical stress within the tumor microenvironment (TME). These conditions continuously expose the cells to pro-inflammatory cytokines, including transforming growth factor-β (TGF-β), interleukin-6 (IL-6), and tumor necrosis factor-α (TNF-α). These signaling molecules work together to activate three critical pathways: nuclear factor-κB (NF-κB), signal transducer and activator of transcription 3 (STAT3), and hypoxia-inducible factor-1α (HIF-1α). These pathways converge on the enhancer region of the ZEB1 gene, forming a transcriptional amplifier that markedly increases ZEB1 expression (8). As a central EMT transcription factor, ZEB1 suppresses E-cadherin through two mechanisms. First, it binds directly to E-box sequences in the E-cadherin promoter to inhibit transcription. Second, it recruits histone deacetylases (HDACs) and DNA methyltransferases (DNMTs), which silence epithelial identity permanently through epigenetic modification (8). Concurrently, Snail and Twist family proteins are upregulated, creating a cooperative network that ensures complete suppression of E-cadherin expression (9, 10).

While these mechanisms are well-established in HNSCC, direct experimental validation of this exact cascade in HNCUP is currently unavailable. Nevertheless, the early metastatic dissemination and occult primary phenotype characteristic of HNCUP are consistent with robust EMT activation, and this cascade provides a biologically plausible framework for understanding HNCUP progression.

**Figure 1:**
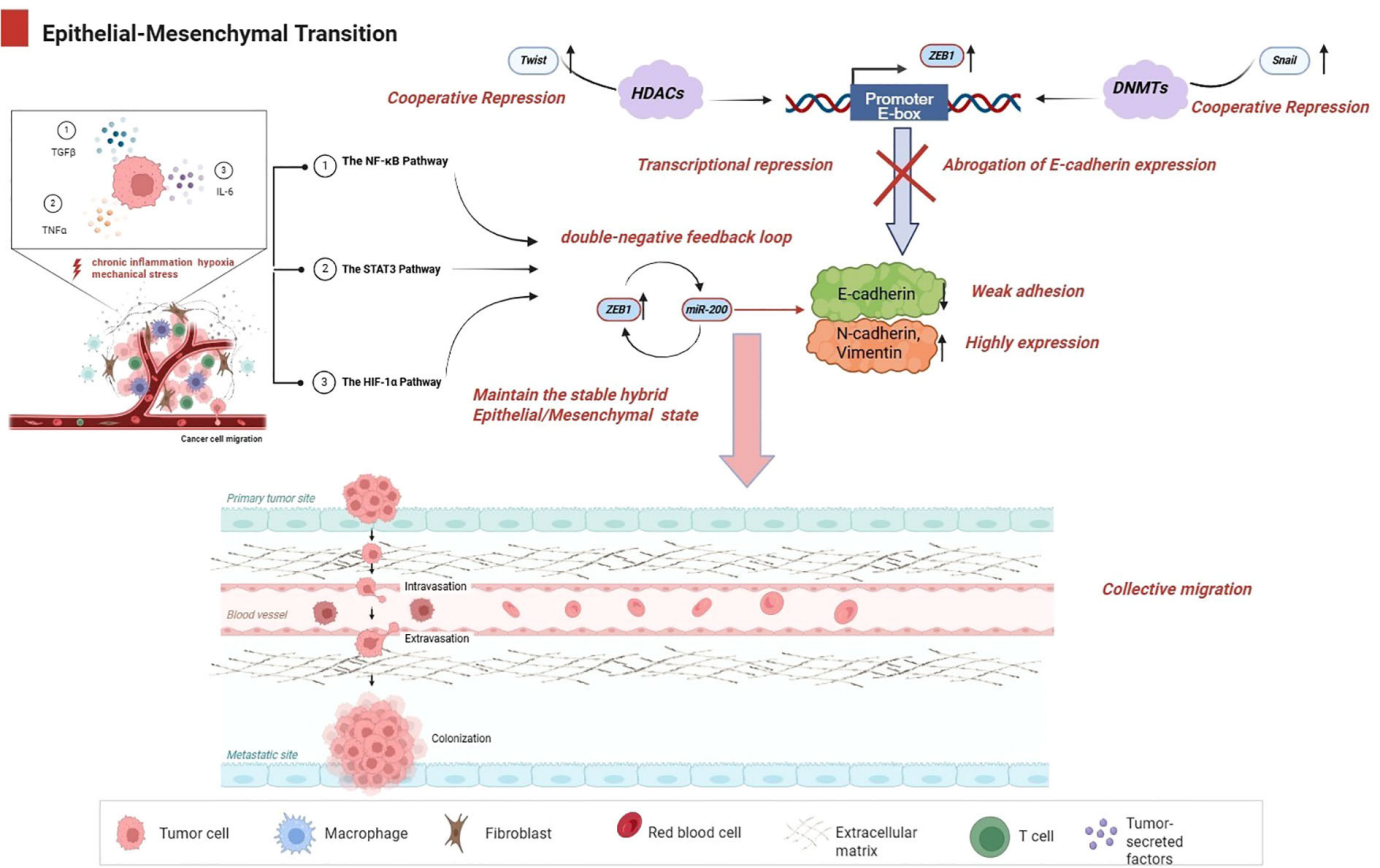
Microenvironment-driven mechanisms of EMT in HNCUP progression. This schematic illustrates the key molecular events governing EMT initiation, maintenance, and early tumor dissemination as established in HNSCC models and extrapolated to HNCUP based on shared biological features of head and neck squamous malignancies. Tumor microenvironmental stressors—including chronic inflammation, hypoxia, and mechanical strain—sustain the release of TGF-β, IL-6, and TNF-α. These cytokines coordinately activate NF-κB, STAT3, and HIF-1α signaling, which converge to upregulate the core EMT transcription factor ZEB1. ZEB1 then silences E-cadherin epigenetically by binding directly to E-box sequences in its promoter and recruiting HDACs and DNMTs. Simultaneously, ZEB1 cooperates with Snail and Twist family proteins to form a multi-factorial repression network that dismantles epithelial identity. A double-negative feedback loop between ZEB1 and miR-200 stabilizes a hybrid epithelial/mesenchymal state characterized by residual E-cadherin alongside elevated N-cadherin and vimentin. This phenotype enables collective migration into lymphatic vessels, generating circulating tumor cell clusters that resist anoikis and facilitate regional occult metastasis. The diagram also depicts relevant cellular components and their secreted factors within the tumor microenvironment that modulate EMT progression. The molecular pathways depicted are derived primarily from HNSCC and pan-cancer studies. While HNCUP shares histopathological and biological features with HNSCC that make EMT-mediated early dissemination a plausible mechanism for its occult primary phenotype, direct experimental validation of this exact cascade in HNCUP is currently unavailable. (Created with BioRender.com).

Notably, EMT in HNCUP commonly manifests as a stable hybrid epithelial/mesenchymal (E/M hybrid) state maintained by a ZEB1/miR-200 double-negative feedback loop. Cancer cells in this state retain partial E-cadherin expression to maintain weak cell-cell junctions while simultaneously upregulating N-cadherin and vimentin. This phenotype enables collective migration, wherein cells detach from the primary site as clusters, penetrate the basement membrane, and invade lymphatic vessels (11). This collective behavior significantly enhances the survival rate of CTCs under hemodynamic shear stress. It also suppresses anoikis—a form of programmed cell death triggered by the loss of extracellular matrix attachment and cell-cell contact-derived survival signals—through intercellular paracrine signaling. These properties represent a critical step in the progression of HNCUP from primary tumor detachment to metastatic colonization (**Figure 1**).

### 2.2 Clonal selection of circulating tumor cells

**Figure 2:**
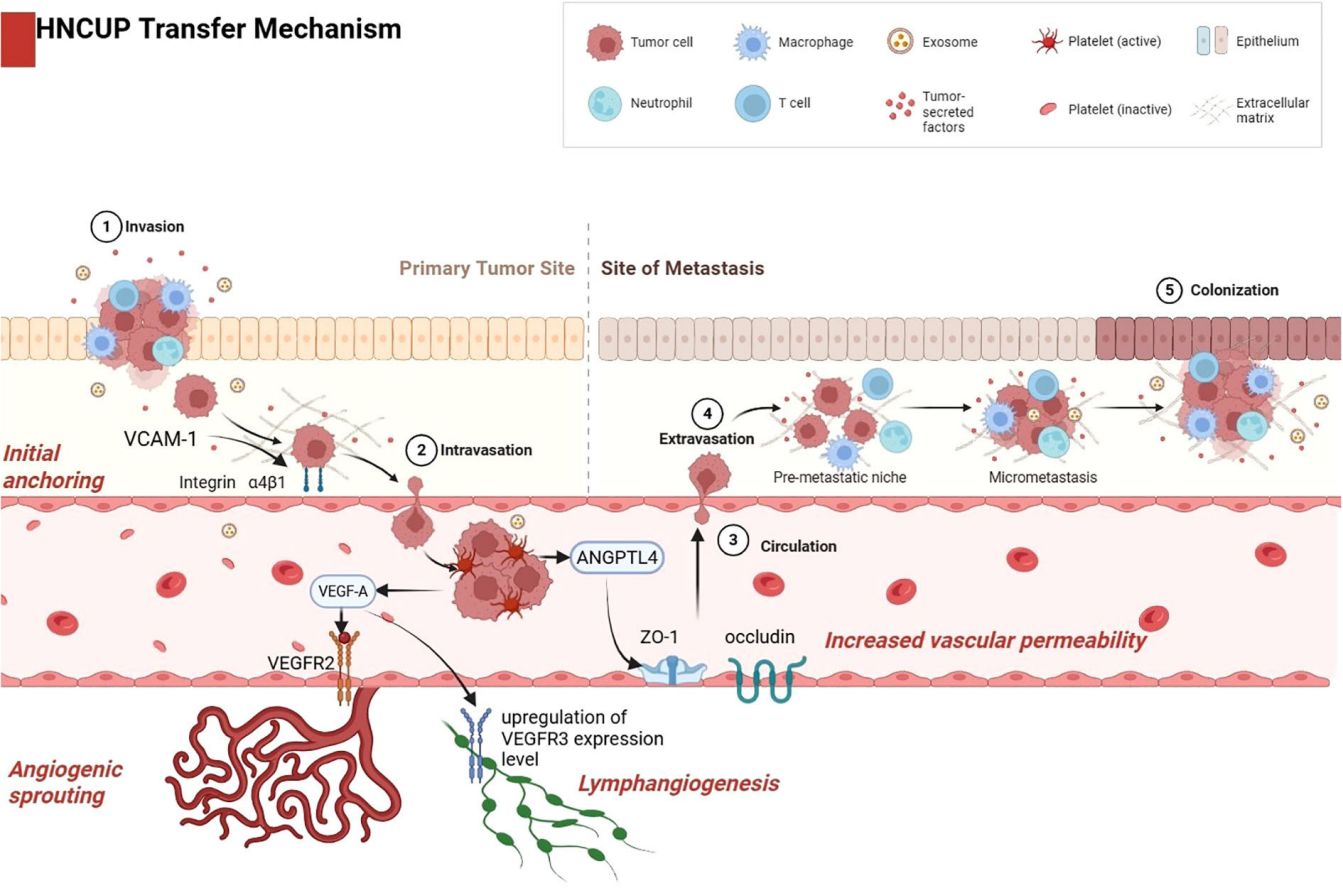
The metastatic cascade and target organ microenvironment remodeling in HNCUP: a seed-and-soil perspective. This schematic illustrates the multi-step process of distant metastasis as conceptualized from general cancer biology and HNSCC studies and proposed as a framework for understanding HNCUP, encompassing invasion, intravasation, circulation, extravasation, and colonization, along with its underlying molecular mechanisms. In preclinical models, CTCs are proposed to function as “seeds.” As demonstrated in HNSCC and other epithelial malignancies, they undergo initial anchoring through specific binding between integrin α4β1 on their surface and VCAM-1 expressed on high endothelial venules in target organs. Subsequently, these CTCs secrete VEGF-A, which activates VEGFR2 and VEGFR3 on endothelial cells to drive angiogenesis and lymphangiogenesis. In experimental models, they release ANGPTL4, which has been shown to downregulate tight junction proteins including ZO-1 and occludin, thereby increasing vascular permeability. These coordinated actions facilitate tumor cell extravasation. Ultimately, the CTCs achieve proliferative colonization in a receptive “soil”—the metastatic niche microenvironment. This schematic represents a conceptual model based on HNSCC and general cancer biology evidence. While the seed-and-soil framework provides a biologically plausible mechanism for understanding the selective metastasis and occult primary phenotype observed in HNCUP, direct experimental confirmation of integrin-mediated CTC anchoring and ANGPTL4-driven vascular permeability in cervical lymph nodes of HNCUP patients has not been established. (Created with BioRender.com).

Based on general cancer biology and HNSCC studies, the “seed and soil” hypothesis provides a conceptual framework for understanding metastasis in HNCUP. This concept posits that successful colonization depends on molecular compatibility between CTCs and the target organ microenvironment. It elegantly explains the paradox of an occult primary tumor coexisting with highly selective metastasis, and delineates a three-phase mechanism comprising initial anchoring, adaptation, and proliferation (12). In preclinical models, CTCs are proposed to function as “seeds.” It has been demonstrated in HNSCC and other epithelial malignancies that integrin family members on CTC surfaces—particularly α4β1— recognize and bind to vascular cell adhesion molecule-1 (VCAM-1), which is highly expressed on stromal cells within cervical lymph nodes and on high endothelial venules (HEVs). This initial anchoring represents the first step in CTC retention at the target organ and resistance to hemodynamic shear stress. Upon successful anchoring, CTCs promptly initiate a paracrine program, secreting multiple pro-angiogenic and pro-lymphangiogenic factors. Among these, vascular endothelial growth factor A (VEGF-A) and angiopoietin-like 4 (ANGPTL4) are the most clinically relevant. VEGF-A activates endothelial VEGFR2 to promote capillary sprouting and simultaneously upregulates VEGFR3 on lymphatic endothelial cells, cooperatively driving lymphatic vessel expansion. In experimental models, ANGPTL4 has been shown to complement this process by suppressing tight junction proteins such as ZO-1 and occludin, thereby increasing vascular permeability and creating space for tumor cell extravasation and stromal invasion (3) (**Figure 2**). While these mechanisms are well-established in preclinical and HNSCC models, direct experimental validation of integrin-mediated CTC anchoring and ANGPTL4-driven vascular permeability in cervical lymph nodes of HNCUP patients is currently unavailable. This framework therefore provides a conceptually plausible model for understanding HNCUP metastatic colonization that requires HNCUP-specific confirmation.

### 2.3 Patterns of cervical lymph node metastasis

The rich lymphatic network of the head and neck provides natural conduits for early tumor dissemination, enabling malignant cells to metastasize to specific nodal groups via regional lymphatic drainage during the initial disease phase. Level II upper deep cervical lymph nodes represent the most common first-echelon metastatic site for malignancies arising from the oral cavity and oropharynx. This phenomenon rests on well-defined anatomical foundations and has been consistently validated through extensive clinical and basic research. It serves as the cornerstone for HNCUP staging, treatment planning, and prognostic assessment. According to international consensus guidelines for cervical lymph node classification, Level II encompasses the upper segment of the internal jugular vein (from skull base to hyoid bone) and primarily receives lymphatic drainage from the oropharynx, tonsils, tongue base, nasopharynx, and portions of the scalp (13). These regions contain abundant submucosal lymphatic plexuses with loose architecture, facilitating ready invasion of malignant cells into lymphatic capillaries and subsequent downstream migration along paths of least resistance. This is particularly evident in oropharyngeal squamous cell carcinoma, where primary lesions frequently arise in lymphoid-rich areas such as tonsillar crypts. The absence of dense connective tissue barriers in these locations permits rapid tumor access to efferent lymphatics and preferential initial drainage to adjacent Level II nodes (3).

**Figure 3:**
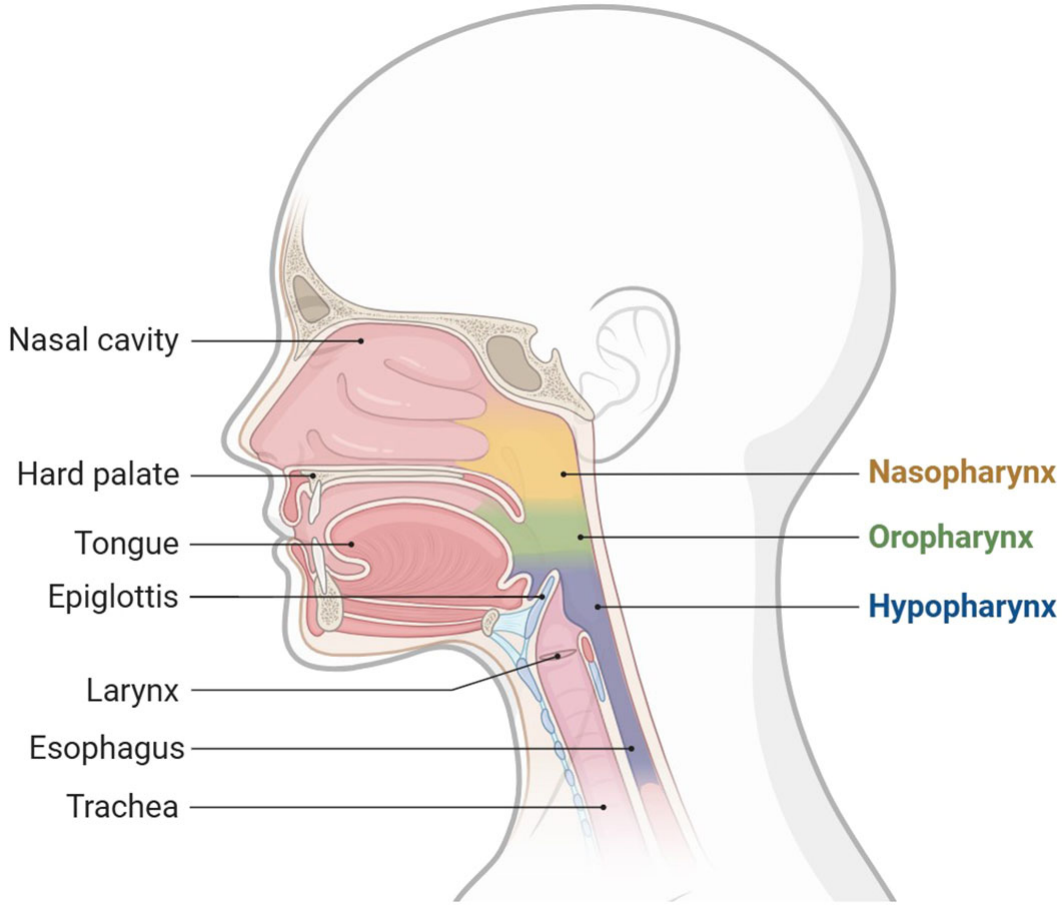
Anatomical origins of primary tumors in HNCUP: a topographic guide. This schematic illustrates the major mucosal and organ sites within the head and neck. Based on established lymphatic drainage patterns, tumors from specific anatomical locations preferentially metastasize to their respective regional lymph nodes, providing essential topographic clues for localizing the occult primary in HNCUP. For example, Level II (upper jugular) nodes represent the most common first-echelon drainage site for oropharyngeal, oral cavity, and nasopharyngeal cancers. Retropharyngeal or Level V involvement strongly suggests a nasopharyngeal primary, whereas Level VI (central compartment) metastasis is characteristic of thyroid carcinoma. Supraclavicular lymphadenopathy warrants evaluation for distant primaries, particularly lung and breast malignancies. (Created with BioRender.com).

Emerging evidence indicates that the specific pattern of involved lymph node groups can provide critical clues to the probable primary site in HNCUP. **Table 1** and **Figure 3** summarize the common metastatic patterns in HNCUP. Oropharyngeal squamous cell carcinoma, including human papillomavirus (HPV)-associated cases, typically metastasizes to Levels II and III, with occasional spread to Level IV. However, tumors from other head and neck sites—including the oral cavity, larynx, hypopharynx, and nasopharynx—may also disseminate to these nodal stations. Retropharyngeal or Level V nodal involvement should raise suspicion for a nasopharyngeal primary. Level IIB lies in proximity to the parotid tail, a site where salivary gland neoplasms may originate. Cutaneous malignancies metastasizing to parotid lymph nodes are also well-documented. Thyroid carcinoma most commonly metastasizes to the central compartment (Level VI), though lateral cervical, supraclavicular, and superior mediastinal nodes may also be affected. Supraclavicular lymphadenopathy warrants consideration of non-head-and-neck primary sites, including the lung, breast, esophagus, and genitourinary tract (1, 14). Metastasis specifically to the left supraclavicular region (the so-called Virchow’s node) should prompt strong suspicion for an infradiaphragmatic primary (15). Across all cases, the palatine tonsils and tongue base represent the most frequently identified primary sites in HNCUP. However, clinical evaluation must also consider rare origins including cutaneous, hematologic, thoracic, and abdominopelvic sites to avoid diagnostic oversight. Under the staging principles for carcinoma of unknown primary outlined in the 8th edition of the Union for International Cancer Control (UICC)/American Joint Committee on Cancer (AJCC) TNM classification, all HNCUP cases are categorized as T0 (16). For Epstein-Barr virus (EBV)-positive and HPV-positive unknown primary carcinomas, N-staging follows the definitions established for nasopharyngeal carcinoma (NPC) and HPV-positive oropharyngeal cancer, respectively, with biological testing required to guide staging assignment (**Table 2**).

**Table 1:** Common primary sites associated with cervical lymph node metastasis by nodal level.

Nodal group	Possible primary tumor sites
Level IA (submental)	Anterior oral cavity, lower lip
Level IB (submandibular)	Oral cavity, anterior nasal cavity, submandibular gland, midfacial face skin
Level II (upper jugular)	Oropharynx, oral cavity, nasopharynx, nasal cavity, larynx, hypopharynx
Level III (mid jugular)	Oropharynx, oral cavity, nasopharynx, larynx, hypopharynx
Level IV (lower jugular)	Oropharynx, larynx, hypopharynx, upper esophagus, thyroid
Level V (posterior triangle)	Nasopharynx, posterior scalp skin, thyroid
Level VI (anterior compartment)	Thyroid, larynx, hypopharynx, upper esophagus
Supraclavicular	Non-head and neck, thyroid
Retropharyngeal	Nasopharynx, posterior pharynx
Parotid	Lateral/upper facial and scalp skin, parotid gland

This table summarizes the anatomical lymphatic drainage patterns and associated primary tumor sites for each cervical nodal level (Levels I-VI, retropharyngeal, parotid, and supraclavicular regions) based on international consensus classification. These correlations, derived from anatomical pathways and clinical epidemiological data, serve as a critical reference for retrograde localization of the occult primary in HNCUP based on the distribution of metastatic lymphadenopathy.

**Table 2:** TNM staging system for HNCUP (based on the 8th edition UICC/AJCC staging rules for carcinoma of unknown primary).

Classification	Significance
Tumor (T)	
T0	No primary site was detected following complete assessment, leading to a T0 classification for the primary tumor in all HNCUP cases
Cervical lymph nodes (N)	
Clinical N classification (cN)	
cNx	Regional lymph node status was not assessable
cN0	No regional LN metastasis
cN1	Single ipsilateral LN, ≤3 cm, no ENE
cN2a	Single ipsilateral LN, 3–6 cm, no ENE
cN2b	Multiple ipsilateral LNs, ≤6 cm, no ENE
cN2c	Bilateral or contralateral LNs, ≤6 cm, no ENE
cN3a	Any LN >6 cm, no ENE
cN3b	Any LN with clinical ENE^a^
Pathologic N classification (pN)	
pNx	Regional lymph node status was not assessable
pN0	No regional LN metastasis
pN1	Single ipsilateral LN, ≤3 cm, no ENE
pN2a	Single ipsilateral LN, ≤3 cm, with ENE^a^, single ipsilateral LN, 3–6 cm, no ENE
pN2b	Multiple ipsilateral LNs, ≤6 cm, no ENE
pN2c	Bilateral or contralateral LNs, ≤6 cm, no ENE
pN3a	Any LN >6 cm, no ENE
pN3b	A single LN >3 cm with pathologic ENE^b^ Any multiple ipsilateral/bilateral/contralateral LN(s) with ENE
Distant metastasis (M)	
Mx	Distant metastasis status was not assessable
M0	No distant metastasis was detected
M1	Distant metastasis was confirmed

LN lymph node, ENE extranodal extension, HNCUP head and neck carcinoma of unknown primary, a Clinical ENE refers to unambiguous clinical/radiological evidence of gross ENE, including dermal involvement, soft tissue invasion with deep fixation or tethering to underlying muscle or adjacent structures, or clinical signs of nerve involvement b Pathologic ENE could be further recorded as ENE mi: microscopic ENE ≤ 0.2 cm beyond nodal capsule; ENE ma: major ENE > 0.2 cm beyond nodal capsule; soft tissue deposit within lymphatic drainage without identifiable LN would be recorded as pN+ and ENE+.

Given the unknown primary site, all HNCUP cases are classified as T0. N-staging (clinical cN and pathological pN) is determined by nodal size, number, laterality, and the presence of ENE. Pathological confirmation of ENE in pN staging provides enhanced prognostic discrimination. M-staging evaluates the presence of distant metastasis. For EBV-positive or HPV-positive HNCUP, N-staging follows site-specific criteria established for NPC and HPV-related oropharyngeal cancer, respectively, underscoring the critical role of biomarkers in precision staging.

In clinical practice, comprehensive analysis of the specific distribution pattern of involved lymph nodes, combined with strict adherence to TNM staging that incorporates extranodal extension (ENE) status and biomarker information (HPV/EBV), provides essential anatomical and biological clues for localizing the occult primary. This integrated approach constitutes the foundation for accurate diagnosis and individualized therapeutic decision-making in HNCUP.

## 3 Diagnostic evaluation of HNCUP

### 3.1 History and physical examination

Patients with HNCUP typically present with a painless, progressively enlarging neck mass as the initial manifestation, accompanied by minimal other symptoms. The disease predominantly affects men aged 50–60 years with limited or no history of tobacco and alcohol use. When conventional evaluation fails to identify the primary tumor, systematic and comprehensive history-taking and physical examination become the cornerstone of the diagnostic pathway, directly revealing the occult primary in up to 53% of cases (14) and guiding subsequent targeted imaging and molecular assessment. Essential history should encompass relevant risk factors including tobacco and alcohol consumption, prior malignancies, skin lesions or cancers, previous radiation exposure, and family cancer history. Physical examination requires a complete head and neck evaluation, including palpation of the palatine and lingual tonsils, and visualization of the nasopharynx, oropharynx, larynx, and hypopharynx using flexible endoscopy (17). Additionally, the cervical region containing malignant lymph nodes provides anatomical clues for primary site localization based on lymphatic drainage patterns (**Table 1**). Studies indicate that 70%-85% of primary tumors in HNCUP are ultimately localized to the Waldeyer ring of the upper aerodigestive tract, particularly the palatine tonsils and tongue base in the oropharynx (17). The deep cryptic architecture of these structures renders early microscopic lesions highly susceptible to missed detection during standard endoscopic examination. Heavy, long-term tobacco and alcohol use are established drivers of conventional head and neck squamous cell carcinoma (HNSCC). In recent years, oropharyngeal cancer associated with high-risk human papillomavirus (HR-HPV), particularly HPV-16, has emerged as a leading etiology of HNCUP, typically affecting younger patients without significant smoking or alcohol history (18). Therefore, detailed sexual history—including specific inquiry regarding oropharyngeal exposure—is essential for assessing HPV-related malignancy risk. Physical examination should extend beyond mere confirmation of the neck mass to encompass systematic, meticulous evaluation of the upper respiratory and digestive tracts, including bilateral inspection and palpation of the oral cavity, oropharynx, hypopharynx, and larynx, supplemented by indirect or direct laryngoscopy. Specific symptom patterns provide critical diagnostic clues: unilateral persistent otalgia (referred pain) suggests ipsilateral oropharyngeal or hypopharyngeal involvement; unexplained hoarseness indicates laryngeal disease; and facial numbness may reflect infraorbital nerve invasion by maxillary sinus or pterygopalatine fossa tumors (19).

In summary, despite advances in modern medicine—including FDG-PET/CT and narrow band imaging (NBI)—no technology can substitute for meticulous history-taking and systematic physical examination. Together, these fundamental clinical skills enhance diagnostic accuracy, prevent unnecessary invasive procedures, and enable individualized therapeutic planning in HNCUP.

### 3.2 Histopathological, immunohistochemical, and molecular analysis of biopsy specimens

Systematic histopathological, immunohistochemical, and molecular analysis of cervical metastasis biopsy specimens represents a cornerstone strategy for primary site localization and individualized therapeutic planning in HNCUP. These complementary approaches progressively narrow the differential diagnosis by advancing from morphological to molecular phenotyping. Histopathological examination serves as the foundation of diagnostic evaluation, with specific tumor subtypes providing critical clues to the probable primary origin. For instance, analysis of hematoxylin and eosin (H&E)-stained sections enables pathologists to confirm histological subtype and assess differentiation. Identification of squamous cell carcinoma initially restricts the search to the upper aerodigestive tract, particularly the tonsillar crypts and tongue base—sites that account for the majority of HNCUP primaries (20).

Building upon this foundation, immunohistochemistry (IHC) functions as a molecular guide to elucidate tumor origin. Using specific antibodies, IHC detects protein markers that characterize tumor lineage. P16 overexpression serves as a robust surrogate marker for HPV infection. A nationwide Danish cohort study demonstrated that p16 testing significantly enhances primary site prediction, with positivity strongly indicating an oropharyngeal origin and favorable overall survival, thereby substantially improving diagnostic precision (21). Additionally, Kühn and colleagues evaluated the prognostic value of podoplanin (D2–40 gene) in HNCUP metastases, demonstrating elevated expression levels that correlate with disease pathogenesis (22).

Molecular analysis is equally essential. EBV genomic DNA and EBV RNA from the Epstein-Barr encoding region (EBER) are strongly associated with NPC. EBER *in situ* hybridization effectively identifies EBV-positive cases, facilitating recognition of NPC-associated HNCUP (23, 24). This molecular “reverse mapping” strategy enables high-probability localization even when the primary tumor is microscopic or completely regressed. MicroRNAs (miRNAs) represent another critical molecular class, established as significant regulators of HNSCC progression, recurrence, metastasis, and survival (25). Barker et al. demonstrated site-specific miRNA expression profiles in tonsillar, tongue base, and nasopharyngeal carcinomas that remain stable in corresponding metastatic lymph nodes, supporting miRNA analysis as a valuable tool for primary site identification in HNCUP (26). Thus, miRNA profiling serves as an additional diagnostic modality for HNCUP evaluation.

### 3.3 Imaging and endoscopic evaluation

#### 3.3.1 Positron emission tomography/computed tomography

18F-fluorodeoxyglucose positron emission tomography (FDG-PET) is widely employed in oncological imaging to assess tissue metabolic activity, distinguish malignant lesions from post-treatment changes, and identify residual viable tumor. This functional imaging modality holds particular value in HNCUP evaluation. In a study by Mani and colleagues, FDG-PET identified putative primary sites in 27 of 52 HNCUP cases, with 24 true positives and 3 false positives, yielding a positive predictive value of 88.9% (27). However, given the potential for false-positive and false-negative findings, FDG-PET results require histopathological confirmation. Orlando et al. retrospectively analyzed disease-free and overall survival in 69 HNCUP patients (1987-2002), demonstrating that FDG-PET achieved high sensitivity (69%) and the highest negative predictive value (87%) for primary site detection (2). These findings establish FDG-PET as a valuable tool for identifying occult primaries after initial conventional workup has failed. A significant limitation of standalone FDG-PET is the absence of anatomical detail—a particular constraint in the complex head and neck region, where patient positioning variability may further compromise diagnostic accuracy. Integration with computed tomography (CT) substantially mitigates these limitations. Kwee and colleagues conducted a systematic review and meta-analysis of 11 studies comprising 433 HNCUP patients who underwent FDG-PET/CT (2005-2007), reporting an overall primary tumor detection rate of 37%, with pooled sensitivity of 84% (95% CI: 78%-88%) and specificity of 84% (95% CI: 78%-89%) (28). Florian et al. directly compared standalone FDG-PET with integrated PET/CT for detecting occult primaries in head and neck carcinoma of unknown primary. Integrated PET/CT demonstrated significantly superior primary site detection rates (55.2% versus 30.8%; p = 0.039) and positive predictive value (93.3% versus 46.1%; p = 0.01) (29). These data indicate that PET/CT offers superior diagnostic performance by fusing CT-derived anatomical precision with FDG-avidity mapping, enabling accurate localization of even minute lesions (30). Therefore, combined FDG-PET/CT represents the preferred imaging approach for primary site localization in HNCUP, offering critical advantages in the anatomically intricate and mobile head and neck region.

Current evidence establishes FDG-PET as a valuable modality in HNCUP management. By detecting the Warburg effect—aerobic glycolysis characterized by preferential glucose conversion to lactate rather than mitochondrial oxidative phosphorylation—FDG-PET enables functional imaging of hypermetabolic lesions, effectively identifying occult primaries missed by conventional evaluation, with favorable sensitivity and negative predictive value. Furthermore, PET/CT significantly enhances detection rates and diagnostic accuracy while reducing false positives and localization errors through integrated anatomical-metabolic assessment, establishing this modality as essential in contemporary HNCUP imaging.

#### 3.3.2 Narrow band imaging

NBI is an advanced endoscopic technique that employs optimized spectral filtering to enhance visualization of mucosal microvasculature and epithelial glandular architecture. By detecting neovascularization and vascular pattern abnormalities within superficial mucosa, NBI provides substantial diagnostic value for localizing occult primary tumors in HNCUP (31, 32). Although PET/CT plays an important role in detecting occult primaries, partial volume effects and other physical limitations may result in missed detection of minute tumors, particularly lesions measuring only a few millimeters in diameter (33). As a critical adjunct to conventional WLI, NBI significantly improves detection rates for occult primaries, particularly in anatomically challenging regions such as the tonsillar fossae, tongue base, pyriform sinuses, and posterior hypopharyngeal wall (34). Multiple studies have demonstrated that NBI-guided targeted biopsy substantially enhances primary site identification. Di Maio and colleagues conducted a meta-analysis of 5 studies comprising 169 HNCUP patients, employing a random-effects model to quantitatively assess NBI performance. Pooled sensitivity was 83% (99% CI: 0.54–0.95), specificity 88% (99% CI: 0.55–0.97), and overall detection rate 35% (99% CI: 0.18–0.53)—all significantly superior to conventional white light endoscopy for identifying minute superficial occult carcinomas (34). Westra et al. prospectively validated these findings, demonstrating a 32% primary site detection rate among WLI-negative/NBI-positive biopsy specimens—lesions that would have been missed with WLI alone—highlighting NBI’s potential for identifying suspicious upper aerodigestive tract lesions in HNCUP (35). In summary, NBI has become integral to standardized diagnostic algorithms for HNCUP. Routine application of enhanced imaging techniques such as NBI during outpatient endoscopy is essential for excluding occult primaries, reducing time to diagnosis, minimizing unnecessary imaging studies, and decreasing healthcare costs and procedural complications.

## 4 Treatment of HNCUP

### 4.1 Surgical management

**Figure 4:**
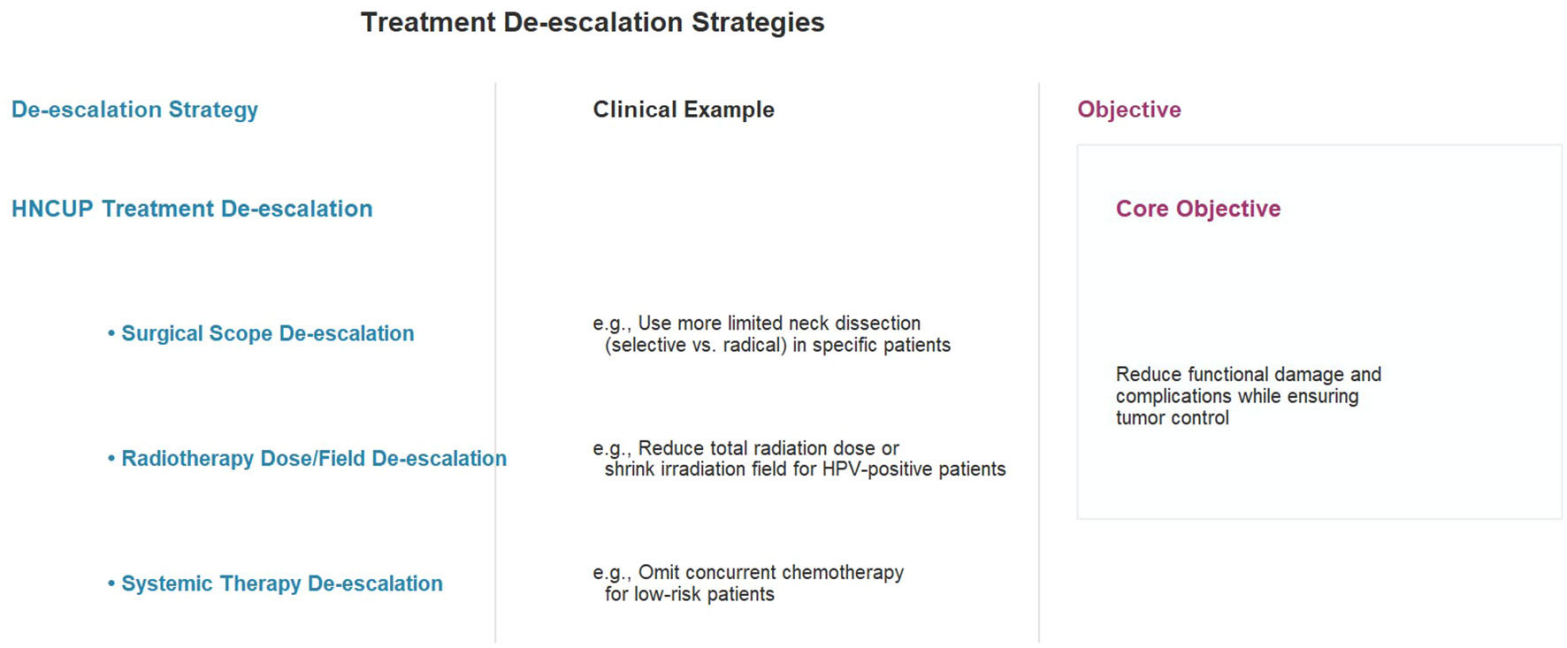
Framework for treatment de-escalation in HNCUP: balancing oncologic control and functional preservation. This schematic illustrates a strategic framework for treatment de-escalation in HNCUP, designed to balance tumor control with functional organ preservation. The framework encompasses three principal dimensions: (1) surgical de-escalation, including selective neck dissection or TORS-guided primary site resection to minimize therapeutic morbidity; (2) radiotherapy de-escalation, such as reduced total dose or target volume contraction for HPV-positive disease; and (3) systemic therapy de-escalation, exemplified by omission of concurrent chemotherapy in low-risk patients. All strategies converge on a unified objective: maximizing treatment-related functional preservation and minimizing long-term complications while maintaining regional tumor control and survival, thereby enabling personalized and precision therapeutic decision-making.

Surgical management in HNCUP extends beyond conventional neck dissection to encompass precise surgical handling of cervical metastatic disease and identification of the occult primary. Transoral robotic surgery (TORS) enables precise resection of suspected primary sites, particularly in the tonsillar fossae and tongue base, and is especially valuable for p16-positive, HPV-related oropharyngeal primaries. A prospective 2-year study from northern India enrolled 18 HNCUP patients who underwent TORS-assisted ipsilateral radical tonsillectomy with tongue base mucosal wedge biopsy, achieving successful resection of putative primary sites with accurate staging and treatment de-escalation (36, 37) (**Figure 4**). This approach utilizes natural orifice access, eliminating external incisions while significantly reducing postoperative dysphagia and speech impairment without compromising oncological radicality. Neck dissection (ND) represents the other principal surgical modality, with the extent of surgery determined by nodal disease burden. For early-stage, limited metastases (clinical N1-N2a), selective neck dissection may suffice for regional control (**Table 1** and **Table 2**). This approach minimizes surgical morbidity. However, advanced stage (N2b-N3) or multilevel nodal involvement typically necessitates more extensive resection. ND serves not only as a critical tool for regional control in advanced head and neck cancer but also provides essential prognostic information to guide adjuvant therapy planning, as established in surgical and radiation oncology literature (38, 39) and consistent with current multidisciplinary guidelines for squamous cell carcinoma of unknown primary in the head and neck (SCCUP) (40).Recent evidence presents conflicting findings: Wallace and colleagues reported improved regional control with ND plus radiotherapy in 179 HNCUP patients (41), whereas a retrospective study of 260 patients found no impact of ND on overall survival (42). The evidence supporting TORS in HNCUP is limited to small prospective cohorts without control arms, precluding definitive conclusions regarding its impact on survival or quality of life when used as primary treatment for occult primaries. The level of evidence for ND extent—selective versus comprehensive—remains similarly constrained, with current recommendations guided by nodal burden and extrapolated from HNSCC data in the absence of HNCUP-specific prospective trials. Notably, the conflicting findings of Wallace et al. and Kamal et al. may reflect heterogeneity in patient selection, era of treatment, and the evolving role of HPV status in prognostication. In summary, contemporary surgical management of HNCUP integrates TORS for identification and resection of occult primaries in the tonsillar fossae and tongue base with ND for precise staging and regional control. This multimodal strategy enables definitive tumor characterization, facilitates personalized treatment planning, reduces unnecessary chemoradiotherapy, and enhances quality of life while maintaining oncological efficacy.

### 4.2 Radiotherapy

Intensity-Modulated Radiation Therapy (IMRT) has become the standard radiation technique for HNCUP, offering highly conformal dose distributions that enable adequate target coverage while significantly reducing radiation exposure to adjacent organs at risk such as the parotid glands, thereby mitigating acute and late toxicities including xerostomia and mucositis.

However, radiotherapy is not universally indicated. Treatment decisions should integrate HPV status (determined by p16 immunohistochemistry), nodal stage, ENE, number and volume of metastatic nodes, and high-risk features including bilateral or superior mediastinal involvement (40). Patients with isolated Level I or VB metastases exhibiting low-risk pathological features (p16-negative, EBV-negative, negative surgical margins, absence of ENE or perineural invasion) or those with unilateral, resectable N1-N2a disease may be candidates for ipsilateral selective neck dissection or limited nodal excision with close postoperative surveillance. High-risk features including ≥3 metastatic nodes, maximum diameter >3 cm, pathologically confirmed ENE, or fixed nodes constitute clear indications for postoperative adjuvant chemoradiotherapy (43–45).

Combined IMRT and concurrent chemotherapy improves locoregional control and overall survival. Shoushtari et al. retrospectively analyzed 27 HNCUP patients treated with IMRT (2002-2008) across N1-N3 stages, demonstrating excellent nodal control, overall survival, and disease-free survival with concurrent chemoradiotherapy in patients with ENE or advanced N2-N3 disease (45). Ghatasheh and colleagues reported 5-year outcomes in 203 HNCUP patients treated with definitive IMRT: local failure 3%, regional failure 14%, distant metastasis 10%, overall survival 79%, and grade ≥3 late toxicity 7% (44). These findings support risk-adapted, reduced-field definitive IMRT as a strategy achieving favorable tumor control with acceptable late toxicity.

The optimal radiation field for HNCUP remains controversial, particularly regarding bilateral elective mucosal irradiation. Historical practice advocated prophylactic irradiation of bilateral pharyngeal mucosa regardless of primary site identification to reduce contralateral recurrence risk. However, this extensive approach may cause unnecessary toxicity. Rodríguez et al. conducted a comprehensive MEDLINE review demonstrating that routine pharyngeal mucosa and bilateral neck irradiation failed to improve survival while increasing treatment-related toxicity. Following comprehensive diagnostic workup, individualized clinical target volume (CTV) delineation based on N stage and involved nodal regions (**Table 2**) is preferred over routine extensive pharyngeal mucosal irradiation (46).

Current evidence supports definitive IMRT with concurrent chemotherapy, with selective field delineation based on nodal stage, ENE, and HPV/p16 status to minimize unnecessary irradiation, reduce toxicity, and optimize the balance between efficacy and quality of life (47).

### 4.3 Systemic chemotherapy

Systemic chemotherapy is indispensable in multimodal therapy for HNCUP. Platinum-based combinations are widely used to reduce metastatic burden and palliate symptoms, though large-scale phase III trials dedicated to this population remain limited. For surgically managed patients with high-risk features—including extensive extranodal extension, positive margins, multiple node involvement, or perineural invasion—postoperative concurrent chemoradiation is strongly recommended, as concurrent chemotherapy (CCRT) enhances radiosensitivity and improves locoregional control (40).

The evidence base guiding these practices is notably constrained. Most chemotherapy data are extrapolated from HNSCC trials or pan-cancer CUP studies. The GEFCAPI 01 phase II trial demonstrated comparable activity of cisplatin-gemcitabine and cisplatin-irinotecan, with response rates near 55% and median survival of approximately 8 months (48). A subsequent meta-analysis found no regimen solidly proven to prolong survival; although platinum and taxane combinations showed favorable point estimates, wide credibility intervals precluded definitive conclusions (49). HNCUP-specific subsets were often underrepresented, and current recommendations rely substantially on expert consensus and HNSCC data extrapolation in the absence of HNCUP-specific prospective trials.

Platinum-paclitaxel doublets are most frequently employed, with platinum-gemcitabine as a viable alternative. Doublet therapy demonstrates superior efficacy compared with single-agent platinum, whereas triplet regimens confer excessive toxicity without proven survival benefit (50). Investigational approaches include selective intra-arterial chemotherapy; Heianna and colleagues reported complete symptom resolution and 3-year locoregional control of 100% in 7 HNCUP patients with fixed bulky disease treated with intra-arterial nedaplatin and docetaxel combined with radiotherapy (51). This approach remains investigational and requires validation in larger studies.

Immune checkpoint inhibitors have reshaped the therapeutic landscape for recurrent or metastatic HNSCC, with implications for HNCUP. The KEYNOTE-048 trial demonstrated improved overall survival with pembrolizumab plus chemotherapy in PD-L1-positive disease (52), and nivolumab showed benefit in platinum-refractory disease (53). Yet these trials predominantly enrolled patients with known primary sites, leaving HNCUP-specific data scarce. Despite this, HPV-associated HNCUP—which constitutes approximately 70% of cases—demonstrates superior survival (3-year rate 94.8% vs. 80.3%), linked to increased DNA damage susceptibility and radiosensitivity (54). These features may enhance checkpoint inhibitor sensitivity, as elevated PD-L1 expression correlates with pembrolizumab efficacy in HNSCC (52). However, this extrapolation requires HNCUP-specific validation.

For recurrent or unresectable disease, prior radiotherapy often precludes re-irradiation due to cumulative toxicity, leaving systemic therapy as the primary modality. Regimens are largely extrapolated from HNSCC methods, including platinum rechallenge, taxane-based therapy, or cetuximab combined with platinum/5-fluorouracil, yet response durations are modest and no prospective data validate these approaches specifically in HNCUP. This uncertainty is consistent with the broader limitations of CUP chemotherapy research (49). Clinical decision-making therefore requires individualized multidisciplinary assessment, weighing performance status, prior therapy exposure, and anticipated benefit against toxicity.

## 5 Conclusions and future directions

The clinical challenge of HNCUP arises from the dual complexities of its primary lesion’s insidious nature and the heterogeneous behavior of metastasis. Current evidence indicates that cervical lymph node dissemination in HNCUP is not a random occurrence but rather a biological process driven by the interplay of EMT-driven phenotypic transition, CTC clonal selection, and organ-specific microenvironmental adaptation. Although Level II upper deep cervical lymph nodes represent the most frequent initial metastatic site, their elevated metastasis rate does not imply anatomical inevitability alone. Rather, their predilection reflects both anatomical drainage patterns and biologically selective colonization. Integration of the specific lymph node region involved, biomarkers such as HPV and EBV, and the TNM staging of HNCUP provides essential clues for primary site localization and informs individualized therapeutic decision-making.

In diagnosis, systematic history-taking and physical examination remain foundational, while modern techniques have substantially improved primary lesion detection. Histopathological analysis, immunohistochemistry targeting p16 and podoplanin, and molecular testing including EBER *in situ* hybridization and miRNA profiling enable reverse tracing of tumor origins. FDG-PET/CT offers high metabolic sensitivity and negative predictive value for occult primary identification, and NBI endoscopy enhances visualization of superficial mucosal blood vessels, thereby increasing the biopsy positivity rate for microcancerous foci observed directly. The multimodal integration of these diagnostic techniques allows for precise identification of concealed primary lesions, establishing a critical foundation for subsequent individualized treatment strategies.

Therapeutic strategies have evolved from traditional extensive radiotherapy and radical neck dissection toward risk-adapted, individualized management guided by precise TNM staging and biomarkers. TORS facilitates minimally invasive resection of suspected primary sites in the tonsillar fossae and tongue base, enabling definitive diagnosis under minimally invasive conditions. The extent of ND is tailored to nodal burden, and IMRT enables conformal dose delivery with reduced toxicity to organs at risk. Platinum-based doublet chemotherapy remains the cornerstone of systemic therapy for advanced or unresectable disease, though evidence specifically derived from HNCUP populations remains limited. Additionally, novel strategies such as selective intra-arterial chemotherapy offer new treatment options for patients with locally advanced HNCUP.

Several limitations persist in the current HNCUP evidence base. The rarity of “true” HNCUP limits prospective validation of biological hypotheses. Most mechanistic insights—including EMT-driven dissemination, CTC clonal selection, and the seed and soil hypothesis—are extrapolated from HNSCC or pan-cancer studies, and direct HNCUP-specific molecular or clinical evidence remains scarce. Emerging technologies—including liquid biopsy, spatial transcriptomics, and AI-assisted radiomics—require prospective clinical integration before routine adoption. Furthermore, the optimal balance between oncologic control and functional preservation in HPV-positive HNCUP remains an active area of investigation.

Addressing these gaps will require coordinated multi-institutional efforts. Future research should harness cutting-edge technologies, such as single-cell sequencing and spatial transcriptomics, to analyze the dynamic processes of EMT, the heterogeneity of CTCs, and the molecular intricacies of their interactions with the TME, with the goal of identifying more specific therapeutic targets. Non-invasive diagnostic and dynamic monitoring techniques, such as liquid biopsy (including ctDNA and exosomes), can be integrated at the diagnostic level. Furthermore, the incorporation of artificial intelligence (AI)-assisted radiomics and pathological image analysis is anticipated to facilitate earlier and more precise identification of primary lesions while minimizing subjective errors. Future treatment strategies for HNCUP represent a critical focus in the design of clinical trials. The efficacy of emerging therapies, including immune checkpoint inhibitors and targeted therapies, for recurrent or metastatic HNCUP remains to be established through prospective studies. Research and clinical management of HNCUP are inherently interdisciplinary, necessitating the collaboration of diverse teams comprising otolaryngology, oncology, radiology, and pathology. Standardized data collection and long-term follow-up, together with shared databases and biobanks, will progressively enhance the understanding of HNCUP natural history. Ultimately, these efforts aim to facilitate precise diagnosis, risk stratification, and individualized treatment for patients with HNCUP.
